# Le syndrome du soleil levant

**DOI:** 10.11604/pamj.2017.26.176.11445

**Published:** 2017-03-29

**Authors:** Napo Abdoulaye, Sidibe Mohamed Kolé

**Affiliations:** 1 Centre Hospitalier Universitaire, Institut d’'Ophtalmologie Tropical de l’'Afrique (CHU-IOTA)Bamako, Boulevard du Peuple, BP 248, Bamako, Mali

**Keywords:** Morning glory, soleil levant, vaisseaux en rayon de roue, Morning glory, sunrise and ships in wheel radius

## Image en médecine

Le syndrome dit Morning glory ou « belle de jour » est une anomalie congénitale rare de la papille par émergence du nerf optique dans l'oeil. Il est parfois associé à des anomalies graves du système nerveux central. Il atteint préférentiellement les filles et le plus souvent unilatérale. Les circonstances de découverte peuvent être un strabisme, une amblyopie, un nystagmus, une leucocorie ou un trouble réfractif. Ce syndrome peut être également associé à des anomalies du système nerveux central, endocriniennes, rénales, respiratoires, ou dans le cadre d'un syndrome de charge. Nous rapportons le cas d'un adolescent de 12 ans, qui a été amené en consultation par ses parents pour une baisse d'acuité visuelle à l'oeil gauche depuis la naissance qui a été objectivé par une perception lumineuse. La fonction visuelle était conservée à l'oeil droit. On notait une image de soleil levant autour de la papille élargit à l'ophtalmoscopie avec des vaisseaux en rayon de roue et un halo pigmenté au sein d'un territoire atrophique à l'oeil gauche aspect de fond l'oeil normal à droite. Il n'y avait pas d'anomalies congénitales associées et une surveillance fut instituée.

**Figure 1:**
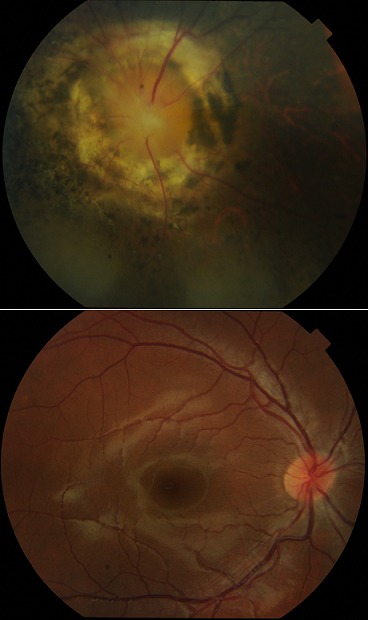
Morning glory

